# Logarithmic Laplacian Prior Based Bayesian Inverse Synthetic Aperture Radar Imaging

**DOI:** 10.3390/s16050611

**Published:** 2016-04-28

**Authors:** Shuanghui Zhang, Yongxiang Liu, Xiang Li, Guoan Bi

**Affiliations:** 1 School of Electronic Science and Engineering, National University of Defense Technology, Changsha 410073, China; lyx_bible@sina.com (Y.L.); lixiang01@vip.sina.com (X.L.); 2 School of Electrical and Electronic Engineering, Nanyang Technological University, Singapore 639798, Singapore; egbi@ntu.edu.sg

**Keywords:** inverse synthetic aperture radar imaging (ISAR), sparse signal recovery, logarithmic Laplacian prior, autofocusing, maximum a posterior (MAP), quasi-Newton method

## Abstract

This paper presents a novel Inverse Synthetic Aperture Radar Imaging (ISAR) algorithm based on a new sparse prior, known as the logarithmic Laplacian prior. The newly proposed logarithmic Laplacian prior has a narrower main lobe with higher tail values than the Laplacian prior, which helps to achieve performance improvement on sparse representation. The logarithmic Laplacian prior is used for ISAR imaging within the Bayesian framework to achieve better focused radar image. In the proposed method of ISAR imaging, the phase errors are jointly estimated based on the minimum entropy criterion to accomplish autofocusing. The maximum *a posterior* (MAP) estimation and the maximum likelihood estimation (MLE) are utilized to estimate the model parameters to avoid manually tuning process. Additionally, the fast Fourier Transform (FFT) and Hadamard product are used to minimize the required computational efficiency. Experimental results based on both simulated and measured data validate that the proposed algorithm outperforms the traditional sparse ISAR imaging algorithms in terms of resolution improvement and noise suppression.

## 1. Introduction

Due to the capability of achieving high resolution images of moving targets (aircrafts, satellites, vessels, *etc.*), the Inverse Aperture Radar Imaging (ISAR) technique has been used for various civil and military applications [1,2,3]. The Range Doppler (RD) algorithm [4], which is supported by the 2D Fast Fourier Transform (FFT), is the most widely applied ISAR imaging algorithm. However, the radar image achieved by the RD algorithm often suffers from high sidelobe and low resolution [5], which hardly meets the requirement of newly developed Automatic Targets Recognition (ATR) techniques. Additionally, complicated motion of targets and low Signal to Noise Ratio (SNR) condition further deteriorate the performance of the RD algorithm [6].

The ISAR image is generally sparse, and only contains a small region of targets with a clear background. Therefore, ISAR imaging can also be achieved by the sparse signal recovery algorithms. Numerous research efforts in this direction have been reported in the past two decades. The sparse signal recovery has led to advanced ISAR imaging methods offering a number of benefits, including the increased resolvability of scatterers, reduced sidelobe, and robustness to limitations in data quality and quantity [7,8,9,10]. The kernel of sparsity-driven ISAR is the sparse signal recovery algorithm, wherein some strategies have been proposed to solve the inverse problems under sparse constraint, such as the basis pursuit (BP) [11], the focal underdetermined system solver (FOCUSS) [12], the orthogonal matching pursuit (OMP) [13], *etc.* These sparse recovery algorithms often suffer from sensitiveness to noise, low computational efficiency or manually tuning of algorithm parameters.

Sparse signal recovery can also be accomplished within the Bayesian framework [14,15]. Compared with the formerly mentioned spare recovery algorithms, the Bayesian sparse signal recovery algorithm performs better in terms of parameter selection, recovery precision, robustness to noise, *etc.* [15]. In recent years, the Bayesian sparse signal recovery algorithms have been introduced to ISAR imaging to improve the quality of radar image [16,17,18,19,20]. The Laplacian prior is utilized in [16,20] to model the ISAR image of the target, and the sparse signal recovery with Laplacian prior is accomplished by the maximum *a posterior* (MAP) estimation and the quasi-Newton method. The ISAR imaging based on the sparse Bayesian leaning (SBL) utilizes the Gaussian scale mixture (GSM) prior to model targets and the variational Bayesian inference [21] based on expectation maximization (EM) to achieve sparse signal recovery [17,18,19].

Compared with the GSM prior, the Laplacian prior enforces the sparsity constraint more heavily, since the distribution of Laplacian prior has a narrower main lobe than the GSM prior, which encourages the signal coefficients closing to zero [22]. Additionally, the log-concavity of Laplacian prior provides a very useful advantage of eliminating local-minima since it leads to unimodal posterior distributions.

In this paper, we propose a novel sparse Bayesian ISAR imaging algorithm with a newly proposed logarithmic Laplacian prior, which is achieved by putting a logarithm on the exponent of the Laplacian prior. Compared to the GSM and Laplacian prior, the proposed logarithmic Laplacian prior has a narrower main lobe and higher tail values, and performs better on sparseness representation. Noting that the ISAR image generally exhibits strong sparse character, the proposed logarithmic Laplacian prior is expected to be more suitable for sparse modeling in ISAR imaging than the widely used GSM and Laplacian prior. Then, the logarithmic Laplacian prior based ISAR image is reconstructed by the MAP estimation, and the phase errors are estimated based on minimum entropy criterion during the iteration of sparse signal recovery. Moreover, the fast Fourier transform (FFT) and Hadamard product are utilized to ensure computational efficiency of the proposed algorithm. Both simulated and measured data based experimental results validate the effectiveness of the prosed method.

This paper is organized as follows. The logarithmic Laplacian prior is defined in Section 2, and the signal model for Bayesian ISAR imaging is presented in Section 3. The Bayesian ISAR imaging based on the logarithmic Laplacian prior, including Bayesian sparse signal recovery, model parameter learning and initialization setting, are derived in Section 4. Experimental results based on both simulated and measured data are illuminated in Section 5, and conclusions are drawn in Section 6.

## 2. Logarithmic Laplacian Prior

The sparse characteristic of the ISAR image makes it possible to achieve ISAR imaging with the sparse Bayesian framework. The image to be reconstructed is usually modeled with some sparse priors, such as the Laplacian prior [16], the Gaussian scale mixture prior [17,18,19], and so on. The probability density function (PDF) of the sparse prior often has a narrower main lobe to promote the reconstructed coefficients to close to zero, and high tail values to guarantee the reconstruction of non-zero coefficients. Generally, the prior with a narrower main lobe and higher tail values can conduct a sparser image. From this perspective, we modify the Laplacian prior to achieve a sparser prior, which is achieved by taking the logarithm on the exponent of the Laplacian distribution, and therefore is called the logarithmic Laplacian prior. Its PDF is defined as:

(1) $$p\left(x\right)=\frac{\lambda }{2}\exp\left[-2\ln\left(\left|x\right|+\lambda \right)\right]$$

where *x* denotes the random variable and *λ* is defined as a scale parameter. Because $\int_{-\infty }^{+\infty }p\left(x\right)dx=1$ is satisfied, *p(x)* is an effective valid PDF.

**Figure 1:**
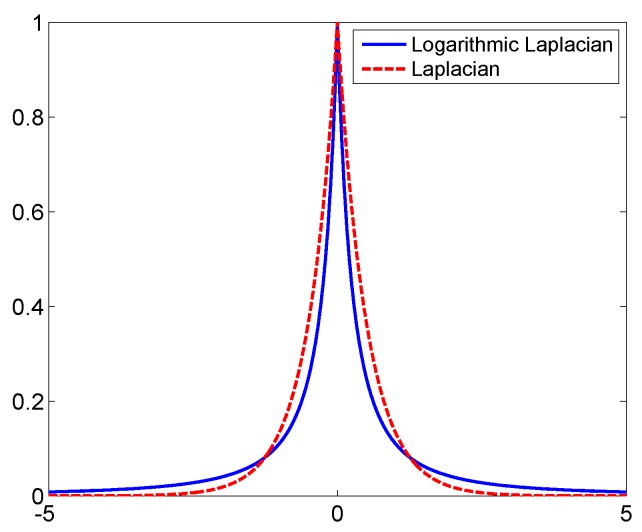
Comparison of the Laplacian and logarithmic Laplacian priors.

Figure 1 shows the comparison between the logarithmic Laplacian prior with λ=0.5 and the Laplacian prior (The Laplace distribution is defined as $p\left(x\right)=\frac{1}{\sqrt{2}\sigma }\exp\left(-\frac{\sqrt{2}}{\sigma }\left|x\right|\right)$) with $\sigma =\frac{1}{\sqrt{2}}a$. It is seen that the logarithmic Laplacian prior has a narrower main lobe with higher tail values than the Laplacian prior to obtain better performance on sparse representation.

## 3. Signal Model with the Logarithmic Laplacian Prior

Supposing the range alignment has been accomplished [23], the process of autofocusing and azimuth compression for ISAR imaging can be modeled as [24,25]:

(2) $$g\left(k,n\right)=\frac{1}{\sqrt{M}}\sum_{m=0}^{M-1}h\left(m,n\right)\exp\left(j\varphi_{m}\right)\exp\left(-j\frac{2\pi }{M}km\right)$$

where $h\left(m,n\right)$, $g\left(k,n\right)$ and $\varphi_{m}$ denote the aligned range profiles, the ISAR image and the phase errors to be compensated, respectively, and *n*, *m* and *k* denote the index of range bin, slow time and Doppler bin, respectively. The aligned range profiles, $h\left(m,n\right)$, can be inversely derived from the ISAR image, $g\left(k,n\right)$, as:

(3) $$h\left(m,n\right)=\frac{1}{\sqrt{M}}\exp\left(-j\varphi_{m}\right)\cdot \sum_{k=0}^{M-1}g\left(k,n\right)\exp\left(j\frac{2\pi }{M}km\right)$$

It can be expressed as:

(4) h=EFg+ε

where **h** and **g** represent the vectorization of $h\left(m,n\right)$ and $g\left(k,n\right)$, respectively, *i.e.*, $h={\left[h\left(0,0\right),\;h\left(1,0\right),\;\cdots ,\;\;h\left(M-1,N-1\right)\right]}^{T}$, $g={\left[g\left(0,0\right),\;g\left(1,0\right),\;\cdots ,\;g\left(M-1,N-1\right)\right]}^{T}$. **E** is a block diagonal phase error matrix as:

(5) $$\begin{array}{c} E={\left[\begin{array}{ccc} e_{M} & & \\ & \ddots & \\ & & e_{M} \end{array}\right]}_{MN\times MN}\;\;\;\;\\ e_{M}={\left[\begin{array}{c} \exp\left(-j\varphi_{0}\right)\\ \;\;\;\;\;\;\;\;\;\;\;\;\;\;\ddots \\ \;\;\;\;\;\;\;\;\;\;\;\;\;\;\exp\left(-j\varphi_{M-1}\right) \end{array}\right]}_{M\times M} \end{array}$$

**F** in Equation (4) represents an MN×MN block diagonal inverse fast Fourier transform (IFFT) matrix, expressed as:

(6) $$\begin{array}{c} F={\left[\begin{array}{ccc} f_{M} & & \\ & \ddots & \\ & & f_{M} \end{array}\right]}_{MN\times MN}\;\\ f_{M}=\frac{1}{\sqrt{M}}{\left[\begin{array}{c} 1\;\;\;\;\;\;1\;\;\;\;\;\;\cdots \;\;\;\;\;1\\ 1\;\;\;\;W_{M}^{-1}\;\;\;\;\cdots \;\;W_{M}^{-\left(M-1\right)}\\ \vdots \;\;\;\;\;\;\;\vdots \;\;\;\;\;\;\;\;\;\;\;\;\;\;\;\;\;\vdots \\ 1\;\;\;W_{M}^{-\left(M-1\right)}\cdots \;\;W_{M}^{-{\left(M-1\right)}^{2}}\; \end{array}\right]}_{M\times M} \end{array}$$

where $W_{M}=\exp\left(-j\frac{2\pi }{M}\right)$. We also have $FF^{H}=F^{H}F=I_{MN}$, where $I_{MN}$ is an MN×MN identity matrix, and ${\left(\cdot \right)}^{H}$ is the conjugate transpose operator.

The additive noise *ε* in Equation (4) is assumed to be zero-mean complex Gaussian distributed:

(7) $$\begin{array}{ccc} p\left(\varepsilon \right) & = & CN\left(\varepsilon |0,\;\alpha I_{MN}\right)\\ & = & \pi^{-MN}\alpha^{-MN}\exp\left(-\alpha^{-1}{∥\varepsilon ∥}_{2}^{2}\right) \end{array}$$

where *α* is the noise variance and ${∥\cdot ∥}_{2}$ represents the $l_{2}$ norm. Then, the likelihood is derived as:

(8) $$\begin{array}{ccc} p\left(h|g;\;\alpha \right) & = & CN\left(h|\mathrm{EFg},\;\alpha I_{MN}\right)\\ & = & \pi^{-MN}\alpha^{-MN}\exp\left(-\alpha^{-1}{∥h-\mathrm{EFg}∥}_{2}^{2}\right) \end{array}$$

The ISAR image, **g**, is assumed to be logarithmic Laplacian distributed:

(9) $$p\left(g;\lambda \right)=\prod_{k=0}^{MN-1}\frac{\lambda }{2}\exp\left[-2\ln\left(\left|g_{k}\right|+\lambda \right)\right]$$

where $g_{k}$ denotes the *k*-th element of **g**.

## 4. Bayesian ISAR Imaging

This section is to derive the Bayesian ISAR imaging based on the logarithmic Laplacian prior.

### 4.1. Sparse Reconstruction of ISAR Image

In this section, the maximum *a posterior* (MAP) estimation based on the quasi-Newton method is utilized to reconstruct the sparse ISAR image. According to the Bayesian theorem, the posterior distribution of the ISAR image, **g**, can be achieved as follows:

(10) $$p\left(g|h;\;\alpha ,\lambda \right)=\frac{p\left(h|g;\;\alpha \right)p\left(g;\lambda \right)}{p\left(h;\alpha ,\lambda \right)}$$

where $p\left(h;\alpha ,\lambda \right)$ is the marginal likelihood which is obtained as:

(11) $$p\left(h;\;\alpha ,\lambda \right)=\int p\left(h|g;\;\alpha \right)p\left(g;\lambda \right)dg$$

Because it is difficult to compute the integral in Equation (11) analytically, the posterior in Equation (10) cannot be derived. Therefore, we utilize the MAP estimation to reconstruct the ISAR image, **g**, as

(12) $$\hat{g}=\arg\max_{g}\left[p\left(g|h;\;\alpha ,\lambda \right)\right]$$

Noting that the marginal likelihood, $p\left(h;\alpha ,\lambda \right)$, is independent of **g**, Equation (12) can be simplified as:

(13) $$\hat{g}=\arg\max_{g}\left[\ln p\left(h|g;\;\alpha \right)+\ln p\left(g;\lambda \right)\right]$$

where the logarithm operator is used for computational convenience. Putting Equations (8) and (9) into Equation (13), and keeping only the terms depending on **g**, we obtain:,

(14) $$\begin{array}{ccc} \hat{g} & = & \arg\max_{g}\left[-MN\ln\pi -MN\ln\alpha -\alpha^{-1}{∥h-\mathrm{EFg}∥}_{2}^{2}+MN\ln\frac{\lambda }{2}-2\sum_{k=0}^{MN-1}\ln\left(\left|g_{k}\right|+\lambda \right)\right]\\ & = & \arg\max_{g}\left[-\alpha^{-1}{∥h-\mathrm{EFg}∥}_{2}^{2}-2\sum_{k=0}^{MN-1}\ln\left(\left|g_{k}\right|+\lambda \right)\right]\\ & = & \arg\min_{g}\left[{∥h-\mathrm{EFg}∥}_{2}^{2}+2\alpha \sum_{k=0}^{MN-1}\ln\left(\left|g_{k}\right|+\lambda \right)\right] \end{array}$$

For convenience, we let $q={∥h-\mathrm{EFg}∥}_{2}^{2}+2\alpha \sum_{k=0}^{MN-1}\ln\left(\left|g_{k}\right|+\lambda \right)$. Noting that **g** is a complex vector, the conjugate gradient of *q* with respect to **g** represents the convergence direction [16], which is derived as:

(15) $$\nabla_{g^{*}}\left(q\right)=H\left(g\right)g-F^{H}E^{H}h$$

where

(16) Hg=IMN+α·diag11gk2+λgkgk2+λgkMN×MN,

where the properties $F^{H}F=I_{MN}$ and $E^{H}E=I_{MN}$ are utilized and $\mathrm{diag}\left[\cdot \right]$ denotes a diagonal matrix whose diagonal elements are defined in the bracket. The Hessian matrix of *q* against **g** should further be derived to utilize the quasi-Newton method. It is seen from Equation (15) that $H\left(g\right)$ resembles a coefficient of **g** and is used to approximate the Hessian matrix of *q* [26]. Then the quasi-Newton iteration is derived as:

(17) $${\hat{g}}^{\left(i+1\right)}={\hat{g}}^{\left(i\right)}-H{\left({\hat{g}}^{\left(i\right)}\right)}^{-1}\nabla_{g^{*}}\left(q\right)$$

where ${\hat{g}}^{\left(i\right)}$ denotes the ISAR image reconstructed in the *i*-th iteration. Substituting Equation (15) into Equation (17), we obtain:

(18) $$\;{\hat{g}}^{\left(i+1\right)}=H{\left({\hat{g}}^{\left(i\right)}\right)}^{-1}F^{H}{\hat{E}}^{\left(i\right)H}h$$

where ${\hat{E}}^{\left(i\right)}$ represents the phase error estimated in the *i*-th iteration.

Furthermore, we utilize the minimum entropy based autofocusing algorithm [24] to estimate the phase error in the iteration, which is achieved as:

(19) $${\hat{\varphi }}_{m}^{\left(i+1\right)}={\hat{\varphi }}_{m}^{\left(i\right)}-{\left.\left[{\left(\frac{\partial^{2}{\tilde{E}}_{g}}{\partial \varphi_{m}^{2}}\right)}^{-1}\frac{\partial {\tilde{E}}_{g}}{\partial \varphi_{m}}\right]\right|}_{\varphi_{m}={\hat{\varphi }}_{m}^{\left(i\right)}}$$

where ${\tilde{E}}_{g}$ represents the simplified entropy [24] of the ISAR image, and $\frac{\partial {\tilde{E}}_{g}}{\partial \varphi_{m}}$ and $\frac{\partial^{2}{\tilde{E}}_{g}}{\partial \varphi_{m}^{2}}$ denote the first and second derivatives of ${\tilde{E}}_{g}$ with respect to $\varphi_{m}$, respectively, given as:

(20) ∂E˜g∂φmφm=φ^mi=-2Imexpjφ^miQmFvectorMNg^ki+2g^kilng^ki*⊙h

(21) ∂2E˜g∂φm2φm=φ^mi=2Reexpjφ^miQmFvectorMNg^ki+2g^kilng^ki*⊙h-2·1MNTvectorMN2+2lng^kihmkmodN2

where Im·, Re·, ⊙, “mod” and * represent the operators for imaginary and real part, respectively, Hadamard product, arithmetical compliment and conjugate operations, respectively. In addition, ${\mathrm{vector}}_{MN}\left(\cdot \right)$ denotes a MN×1 vector. $Q_{m}=\left[0,\cdots ,0,\underset{mN}{1},\cdots ,\underset{\left(m+1\right)N-1}{1},0,\cdots ,0\right]$, and $1_{MN}^{}\;=\;{\left[1,\cdots ,1\right]}_{MN}^{T}$, $h_{j}\left(i\right)\;=\;h_{jN+i}$.

Equations (18) and (19) are iterated until convergence is reached, which can be judged by:

(22) $$\frac{{∥{\hat{g}}^{\left(i+1\right)}-{\hat{g}}^{\left(i\right)}∥}_{2}}{{∥{\hat{g}}^{\left(i\right)}∥}_{2}}\leq \mu \;$$

where *μ* denotes the expected precision. It should be noticed that the computational burden of updating Equation (18) would be intolerable since it needs to inverse the Hessian matrix, $H\left({\hat{g}}^{\left(i\right)}\right)$, which has a dimension of MN×MN. Noting that $H\left(g\right)$ derived in Equation (16) is a diagonal matrix, its inverse matrix is Hg^i-1=diagg^ki2+λg^kig^ki2+λg^kiα+g^ki2+λg^kiα+g^ki2+λg^kiMN×MN. Therefore, Equation (18) is equivalent to multiplying each element of the ISAR image by a coefficient, which can be accomplished by the Hadamard product . Additionally, FFT operation with respect to the slow time can be utilized to achieve the multiplication of $F^{H}$. With these efficient calculations, the computational efficiency can be largely improved.

### 4.2. Model Parameters Learning

The model parameters, including the noise variance, *α*, and the scale parameter, *λ*, should also be estimated to put the proposed Bayesian ISAR imaging based on logarithmic Laplacian prior into practice. Firstly, we use the maximum likelihood (ML) estimation method to estimate the noise variance, *α*, which is derived as:

(23) $$\hat{\alpha }=\arg\max_{\alpha }\left[\ln p\left(h|g;\;\alpha \right)\right]$$

where the likelihood, $p\left(h|g;\;\alpha \right)$, is given in Equation (8). The derivative of $\ln p\left(h|g;\;\alpha \right)$ with respect to *α* is obtained as:

(24) $$\frac{\partial \ln p\left(h|g;\;\alpha \right)}{\partial \alpha }=-\frac{MN}{\alpha }+\frac{1}{\alpha^{2}}{∥h-\mathrm{EFg}∥}_{2}^{2}$$

Setting it to zeros, the noise variance, *α*, is estimated as

(25) $${\hat{\alpha }}^{\left(i+1\right)}=\frac{1}{MN}{∥h-{\hat{E}}^{\left(i\right)}F{\hat{g}}^{\left(i\right)}∥}_{2}^{2}$$

where ${\hat{\alpha }}^{\left(i\right)}$ represents the noise variance estimated in the *i*-th iteration. ${\hat{E}}^{\left(i\right)}$ and ${\hat{g}}^{\left(i\right)}$ are achieved by Equations (18) and (19), respectively. Furthermore, the MAP method is utilized to estimate the scale parameter, *λ*, which is given as:

(26) $$\begin{array}{ccc} \hat{\lambda } & = & \arg\max_{\lambda }\left\{\ln\left[p\left(h|g;\;\alpha \right)p\left(g;\lambda \right)\right]\right\}\\ & = & \arg\max_{\lambda }\left[\ln p\left(g;\lambda \right)\right] \end{array}$$

The derivative of $\ln p\left(g;\lambda \right)$ with respect to *λ* is derived as:

(27) $$\frac{\partial \ln p\left(g;\lambda \right)}{\partial \lambda }=\frac{MN}{\lambda }-2\sum_{k=0}^{MN-1}\frac{1}{\left|g_{k}\right|+\lambda }$$

Setting it to zero, we obtain:

(28) $$\lambda^{\left(i+1\right)}=\frac{MN}{2}{\left(\sum_{k=0}^{MN-1}\frac{1}{\left|g_{k}^{\left(i\right)}\right|+\lambda^{\left(i\right)}}\right)}^{-1}$$

### 4.3. Initialization

This subsection presents the initialization of the proposed algorithm, which is significant for its practical implementation.

Firstly, we utilize the Doppler centroid-based autofocusing method (DCA) to initialize the phase error [27], which is implemented efficiently but suffers from low estimation precision. The initialization of the ISAR image, **g**, can be achieved by the traditional RD imaging as

(29) $${\hat{g}}^{\left(0\right)}=F^{-1}{\hat{E}}^{\left(0\right)-1}h=F^{H}{\hat{E}}^{\left(0\right)H}h$$

where ${\hat{g}}^{\left(0\right)}$ and ${\hat{E}}^{\left(0\right)}$ denote the initial ISAR image and the phase errors, respectively. However, it should be noticed that ${\hat{g}}^{\left(1\right)}={\hat{g}}^{\left(0\right)}$ when Equation (29) is utilized to initialize **g**, which can be derived by combining Equations (18), (25) and (29). It means the initialization with Equation (29) will lead to a local minimal located at ${\hat{g}}^{\left(0\right)}$. In order to avoid this, it is multiplied by a coefficient, $\frac{1}{\sqrt{M}}$, as:

(30) $${\hat{g}}^{\left(0\right)}=\frac{1}{\sqrt{M}}F^{H}{\hat{E}}^{\left(0\right)H}h$$

**Figure 2:**
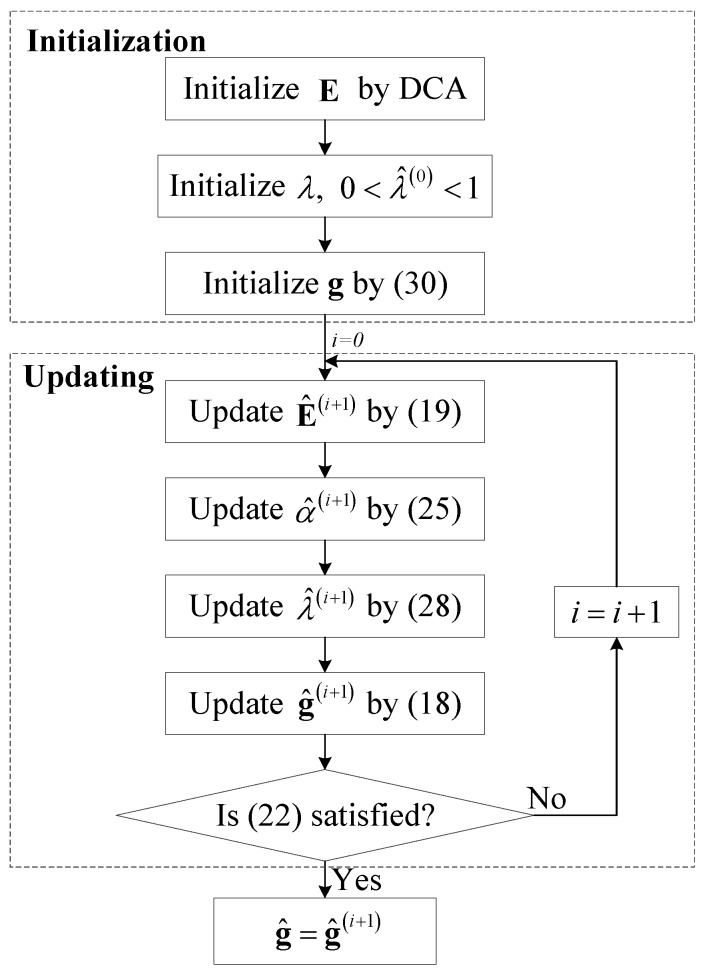
Flow chart of the logarithmic Laplacian prior based Bayesian inverse synthetic aperture radar (ISAR) imaging algorithm.

Finally, the initialization of *λ* can be set in the range of 0 to 1. The flow chart of the logarithmic Laplacian prior based Bayesian ISAR imaging is given in Figure 2. After initialization, Equations (18), (19), (25) and (28) are iterated until convergence is reached, which is evaluated by Equation (22).

## 5. Experimental Results

In this section, experimental results based on both simulated and measured data are analyzed to compare the performance of the proposed logarithmic Laplacian prior based Bayesian (LLB) ISAR imaging algorithm with those of the Laplacian prior based Bayesian (LB) ISAR imaging algorithm [16] and the minimum entropy based on RD (ME-RD) algorithm [24]. The experimental data include the simulated data of a small-sized battleplane (Mig-25), and the measured data of a medium-sized commercial aircraft (Yak-42).

### 5.1. Data Set 1: Simulated Data of Mig-25

The simulated Mig-25 is composed of 120 scatterers of the same scattering intensity [9]. The radar transmits the stepped frequency signal with a center frequency of 9 GHz, a bandwidth of 512 MHz, and a pulse repetition frequency of 15 kHz. The number of the stepped frequencies in a sweep is 64, and the number of sweeps is 512. The Keystone transform [28] and the phase cancellation method [29] are utilized to compensate the migration through range cells and Doppler cells, respectively.

**Figure 3:**
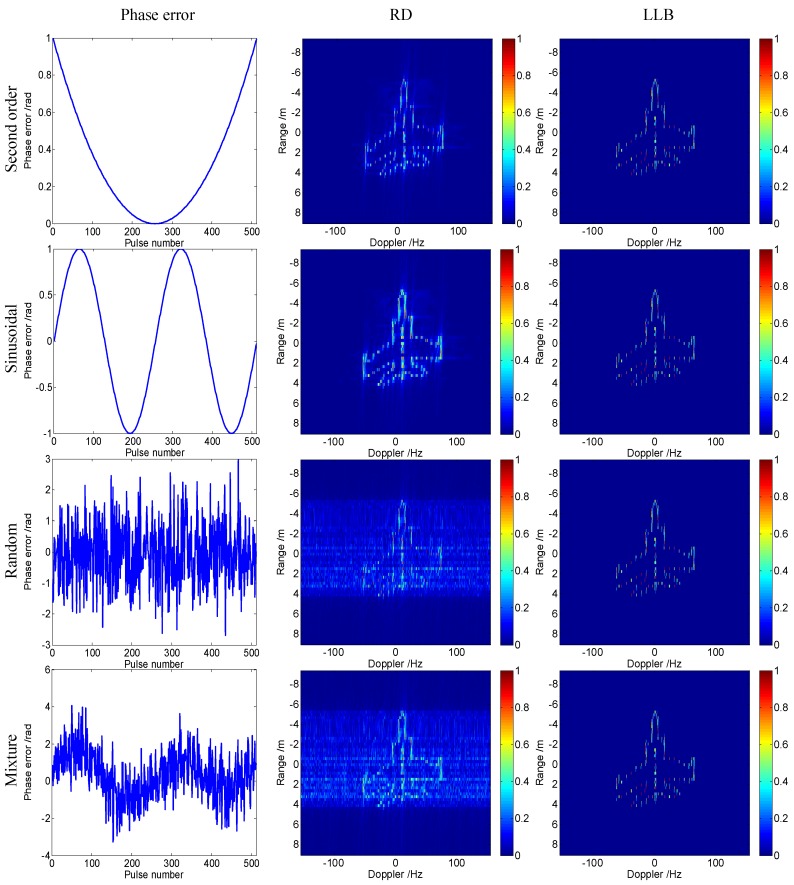
Imagery results of Mig-25 with different phase errors.

Firstly, four types of phase errors, including the second order, the sinusoinal, the random error, and their mixture, are added to the range profiles to testify the validity of LLB for different phase errors. The four phase errors are given in the first column of Figure 3. The terminal parameter, *μ*, in Equation (22), and the initial scale parameter, *λ*, in Equation (9) are set as 0.005 and 1, respectively. Figure 3 gives the imagery results of RD and the proposed LLB. It is seen that the results of RD are defocused for the cases of random and mixture phase errors. In contrast, the proposed LLB obtains well-focused images in any cases, which validates its performance is not affected by the type of phase errors.

**Figure 4:**
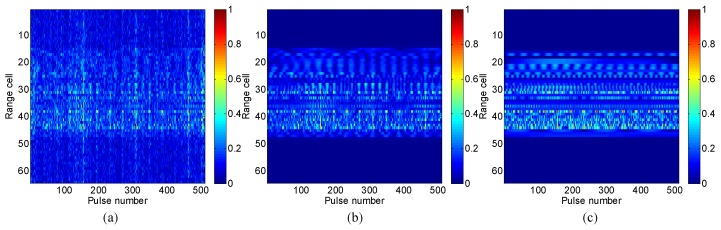
Range profiles of Mig-25 with SNR = 0 dB: (**a**) original; (**b**) the Laplacian prior based Bayesian (LB) method; (**c**) the logarithmic Laplacian prior based Bayesian (LLB) method.

Let us next add the complex Gaussian noise to the radar echo to simulate the noise environment, so as to validate the effectiveness of LLB under low signal to noise ratio (SNR) conditions. Each pulse of the radar echo is added with noise separately, because the noises in different pulses are independent with each other in the real radar system. Figure 4 shows the original range profile and those reconstructed by LB and the proposed LLB with *SNR* = 0 dB, respectively. It is seen that both LB and LLB obtain clear range profiles with noise largely suppressed, and LLB performs relatively better.

**Figure 5:**
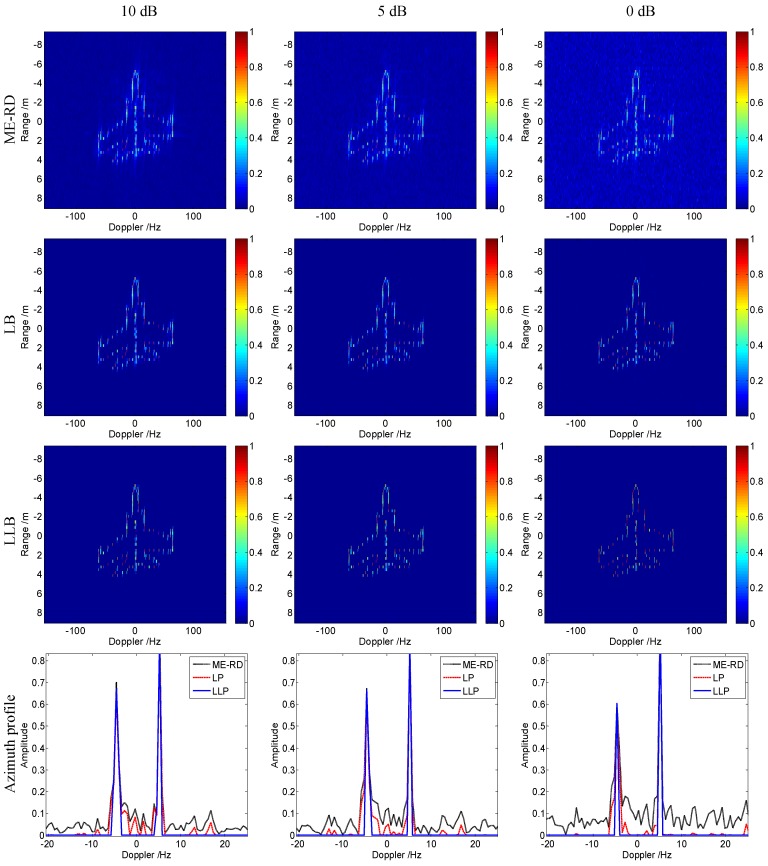
Imagery results of Mig-25 under different signal to noise ratio (SNR) conditions.

Figure 5 shows the normalized imagery results of Mig-25 achieved by ME-RD, LB and LLB with *SNR* = 10 dB, 5 dB and 0 dB, respectively. The threshold is set as 0.005 for both algorithms. As given in the first row of Figure 5, the results of ME-RD are much noisy and affected by high sidelobes. Compared with ME-RD, LB obtains relatively better focused and less noisy images shown in the second row of Figure 5. However, it is still affected by sidelobes and noise. The proposed LLB achieves the best images with little effect of sidelobes and noise under all these SNR conditions, which are given in the third row of Figure 5, which validates the effectiveness of the proposed LLB. The fourth row of Figure 5 gives the azimuth profiles in the 22-th range cell of the image results obtained by these three algorithms. It is seen that the proposed LLB achieves the azimuth profiles with the highest resolution and the lowest noise floor when compared with ME-RD and LB.

**Figure 6:**
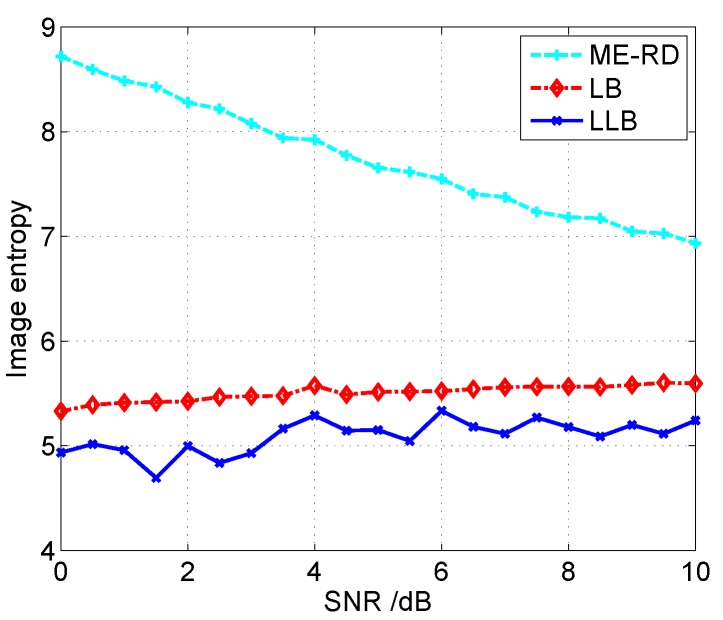
Comparison of image entropy curves *versus* SNR for different algorithms.

Let us use the image entropy [24] to quantitatively compare the performances of these three algorithms, and lower image entropy generally indicates better focused image. Figure 6 gives the image entropy curves *versus* SNR obtained by different algorithms. It is seen that the proposed LLB obtains lower image entropy than ME-RD and LB under all these SNR conditions, and, therefore, it achieves better focused images compared with ME-RD and LB. Additionally, it is noticed that the curve of ME-RD is decreasing, while those of LB and LLB are flat, which indicates the Bayesian ISAR imaging algorithms are more robust to noise than ME-RD.

### 5.2. Data Set 2: Real Measured Data of Yak-42

Real measured data of Yak-42 [7,30] is utilized to further analyze the performance of the proposed algorithm. Yak-42 is a twin engine commercial aircraft with a size of 36 m × 35 m, and during the interval of ISAR imaging, it is flying smoothly at an approximate speed of 380 km/h. The radar transmits the signal with a center frequency of 5.52 GHz and a bandwidth of 400 MHz. The numbers of samples and sweeps are both 256. The original SNR is 15.2 dB, and it is adjusted by additional complex Gaussian noise.

**Figure 7:**
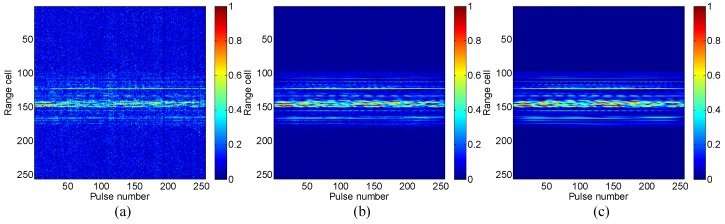
Range profiles of Yak-42 with SNR = 0 dB: (**a**) original; (**b**) LB; (**c**) LLB.

Figure 7 shows the comparison of the original range profile and those obtained by LB and LLB with *SNR* = 0 dB, respectively. It is seen that, compared with LB, the proposed LLB obtains clearer range profile with lower noise floor, which indicates it performs better on noise reduction than LB.

**Figure 8:**
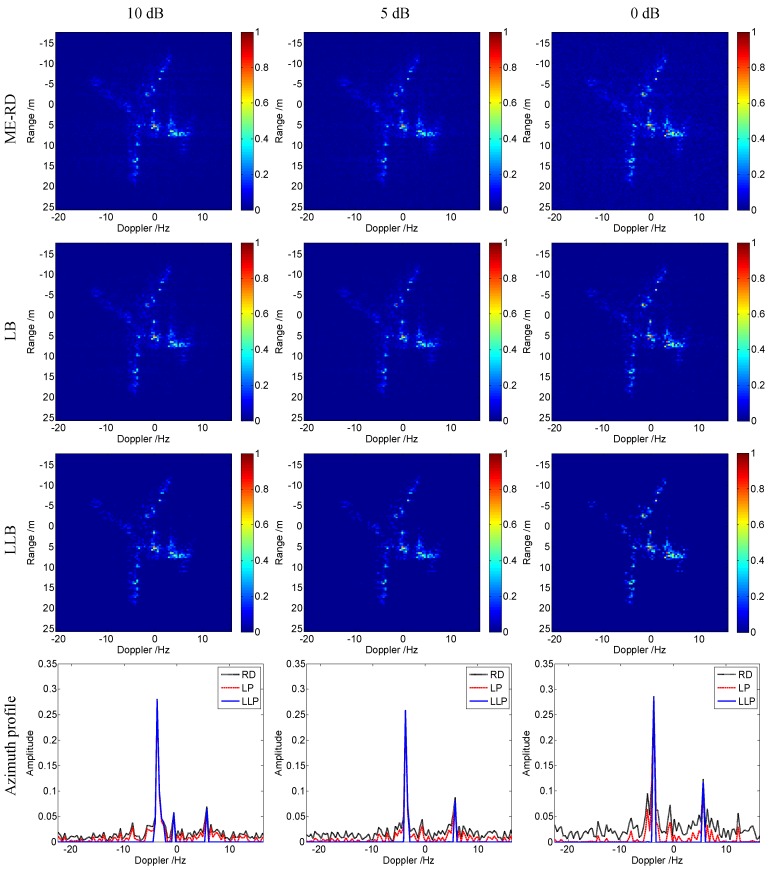
Imagery results of Yak-42 under different SNR conditions.

Figure 8 shows the ISAR imagery results obtained by these algorithms under different SNR conditions. Compared with ME-RD and LB, the proposed LLB obtains the best focused images with the clearest background. Additionally, it should be noticed that the images obtained by LLB for lower SNR are sparser, because the weighted matrix, $H{\left({\hat{g}}^{\left(i\right)}\right)}^{-1}$, in Equation (18), is inversely proportional to the noise variance, *α*, and stronger noise induces smaller weighted coefficient, which broadens the gap between the weighted coefficients of the strong and weak scatterers, and makes the obtained image sparser. Furthermore, the azimuth profiles in the 154-the range cell of the images obtained by different algorithms are given in the fourth row of Figure 8 to compare the algorithms performances more clearly. It is seen that, compared with ME-RD and LB, the proposed LLB achieves the azimuth profiles with narrower peaks and clearer background, which indicates better performances on side lobe suppression and noise reduction, respectively.

**Figure 9:**
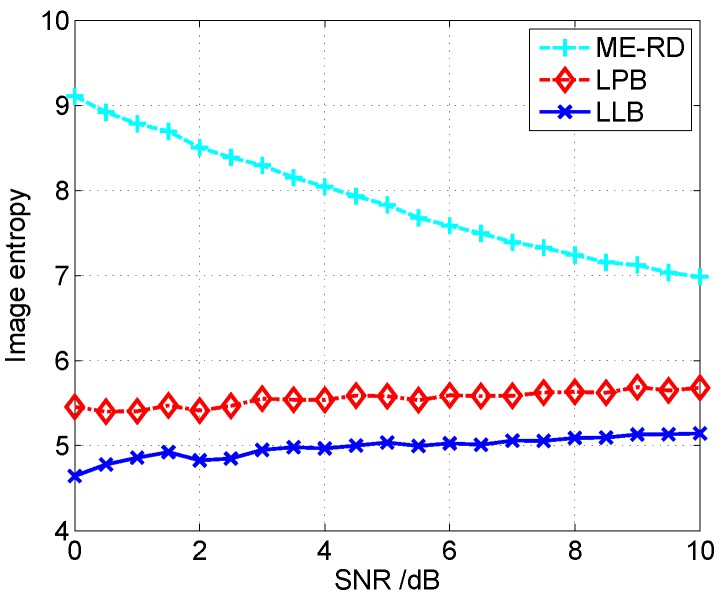
Comparison of image entropy curves *versus* SNR for different algorithms.

**Figure 10:**
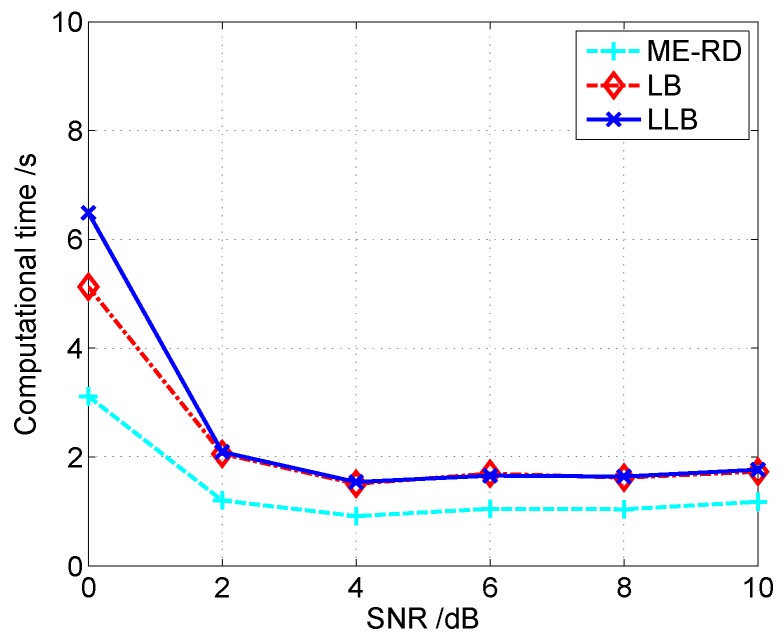
Comparison of computational time *versus* SNR for different algorithms.

Figure 9 shows the image entropy curves *versus* SNR achieved by three algorithms. It is seen that the proposed LLB obtains the lowest image entropy under any SNR conditions, which further confirms its effectiveness. The computational time of three algorithms under different SNR conditions is shown as Figure 10. It shows that the computational burden of the proposed LLB is comparable with that of LB, which is slightly heavier than that of ME-RD.

**Figure 11:**
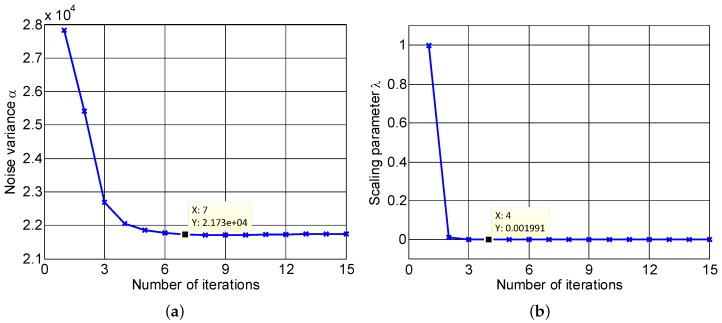
Update of model parameters: (**a**) noise variance *α*; (**b**) scale parameter *λ*.

Last but not least, the parameter updating process of the proposed LLB, including the noise variance, *α*, and the scaling parameter, *λ*, is presented to confirm the effectiveness of learning model of LLB. Figure 11a,b show the converging curves of *α* and *λ*, respectively. It is seen that the noise variance, *α*, converges at $2.173\times 10^{4}$ within seven iterations, and the scaling parameter, *λ*, converges at 0.001991 within four iterations, which confirms the effectiveness of the updating rules given in Equations (25) and (28). Additionally, it should be noticed that the scaling parameter, *λ*, falls to a small value after one iteration, and the small value of *λ* conducts a spiky PDF with a narrow peak and heavy tail values, which is beneficial to the sparse image reconstruction and helps to achieve a well-focused ISAR image within several iterations.

## 6. Conclusions

A new sparse prior called the logarithmic Laplacian prior is presented to conduct a better performance on sparse representation than the Laplacian prior. Then, a novel Bayesian ISAR imaging method based on the logarithmic Laplacian prior is proposed, in which the ISAR image is reconstructed by the MAP estimation based on the quasi-Newton method, and the autofocusing is accomplished based on the minimum entropy criterion. Experimental results based on both simulated and measured data confirm that the proposed algorithm performs better than the Laplacian prior based Bayesian ISAR imaging on sidelobe suppression and noise reduction. Bayesian ISAR imaging based on the logarithmic Laplacian prior for the targets with the complex motion will be the next focus of our research.
